# To disclose, or not to disclose? Perspectives of clinical genomics professionals toward returning incidental findings from genomic research

**DOI:** 10.1186/s12910-021-00670-y

**Published:** 2021-07-27

**Authors:** Isamme AlFayyad, Mohamad Al-Tannir, Amani Abu-Shaheen, Saleh AlGhamdi

**Affiliations:** 1 Research Center, King Fahad Medical City, P.O. Box. 59046, Riyadh, 11525 Saudi Arabia; 2 College of Medicine, Prince Sattam Bin Abdulaziz University, Riyadh, Saudi Arabia

**Keywords:** Incidental findings, Disclosure, Genomic research, Attitudes, Perception, Barriers, Saudi Arabia

## Abstract

**Background:**

Clinical genomic professionals are increasingly facing decisions about returning incidental findings (IFs) from genetic research. Although previous studies have shown that research participants are interested in receiving IFs, yet there has been an argument about the extent of researcher obligation to return IFs. We aimed in this study to explore the perspectives of clinical genomics professionals toward returning incidental findings from genomic research.

**Methods:**

We conducted a national survey of a sample (n = 113) of clinical genomic professionals using a convenient sampling. A self-administered questionnaire was used to explore their attitudes toward disclosure of IFs, their perception of the duties to return IFs and identifying the barriers for disclosure of IFs. A descriptive analysis was employed to describe participants' responses.

**Results:**

Sixty-five (57.5%) respondents had faced IFs in their practice and 31 (27.4%) were not comfortable in discussing IFs with their research subjects. Less than one-third of the respondents reported the availability of guidelines governing IFs. The majority 84 (80%) and 69 (62.7%) of the study participants indicated they would return the IFs if the risk of disease threat ≥ 50% and 6–49%, respectively and 36 (31.9%) reported they have no obligation to return IFs.

**Conclusion:**

Clinical genomics professionals have positive attitudes and perceptions toward the returning IFs from genomic research, yet some revealed no duty to do so. Detailed guidelines must be established to provide insights into how genomics professionals should be handled IFs.

**Supplementary Information:**

The online version contains supplementary material available at 10.1186/s12910-021-00670-y.

## Background

The innovation of new technology in the field of genomics has led to redefining medicine and information-empowerment. Genomics is empowering clinicians due to its preventive and predictive capabilities in the characterization and diagnosis of a wide variety of medical conditions [1]. In genomic research, the extent of data generated is indeterminate and a significant gain, yet it is likely to discover unrelated or incidental genomic data outside the scope of the research objective.

Incidental findings (IFs) are “findings concerning an individual research participant that has potential health or reproductive importance and is discovered in the course of conducting research but is beyond the aims of the study” [2]. Incidental findings are widely controversial in genomic research and raise ethical and legal concerns. The controversy originates from the query whether IFs should be returned to research subjects—and if so, which kind of data are to be revealed and by whom. No doubt many reasons add further layers of complexity to the debate surrounding the disclosure of IFs, like misattributed paternity and clinically actionable findings [3]. In genomic research, IFs may take the form of discovering an individual genetic variant for a particular disease or increased susceptibility to disease. Moreover, it may reveal “extra” and sometimes unwanted information about the family of research subject [2].

Several studies revealed that many research participants want genomic IFs returned to them regardless of its clinical significance or “actionability” [4–7]. Returning clinically significant data, they conclude, would demonstrate the researcher's concern for the research subject's welfare and autonomy and strengthens the fiduciary relationship between the researcher and the research subject. However, some scholars argue that researchers, unlike physicians, are not necessarily required to act for the health benefits of research participants. Unlike physicians who have a duty to follow up on a patient's health, there exists no relationship between researcher and research volunteers similar to doctor/patient relationship in the clinical context [8–10].

Nevertheless, other scholars disagree that researchers do not have a general duty to return incidental findings [11, 12]. According to these scholars, research participants have a right to be informed about these findings given that certain conditions are fulfilled. They proposed five criteria, for the general duty to return incidental findings:

- The findings are analytically valid
- Returning them to the donor comports with applicable law
- The donor has been offered that option of consenting to return of individual findings and has opted to receive them
- The findings reveal an established and substantial risk of (A) a serious health condition, or (B) a serious condition of reproductive importance
- The findings are clinically actionable

Several guidelines have been published on the return of IFs in genomic studies. In the realm of clinical sequencing, the American College of Medical Genetics and Genomics (ACMG) released a recommendation for clinical diagnostic laboratories to return medically actionable secondary findings to all patients regardless of patient preference [1]. This recommendation was later updated to include an option for patients to opt-out of receiving these results. However, controversy still exists around these and other guidelines and the extent to which they should be followed in research settings. In the research setting, similar conclusions were reached by the National Heart, Lung, and Blood Institute (NHLBI) Working Group on Reporting Genetic Results in Research Studies, which released recommendations about the return of genetic results from research studies [13]. They stated that genetic results should be returned to participants when the associated risk for the disease is significant, the disease itself is associated with significant morbidity, and there are interventions available to mitigate the course of the disease. These guidelines imply the existence of an obligation of researchers to return research results that meet these criteria.

Despite the multitude of existing pieces of literature, no literature has been captured in our systematic literature review assessing the clinical genomics professionals toward IFs disclosure in Saudi Arabia. Therefore, we aimed in this study to explore the attitudes of clinical genomics professionals toward disclosure of IFs, their perception of the duties to return IFs, and identifying the barriers for disclosure of IFs.

## Methods

This study involved the administration of online and paper-based self-administered questionnaire using a cross-sectional study design. Eligible clinical genomics professionals were identified through the Saudi Society of Medical Genetics membership database and through searching the genomic scientists working in the Saudi health care institutions and academic universities. Clinical genomic professionals involved in genomic research and testing were recruited using convenient sampling technique. Genomic professionals are clinicians who generate differential diagnoses, prescribe treatment for patients with illnesses that result from genetic anomalies, and council them. Participants were contacted up to three times to participate in the study. An initial and two follow-up emails (for non-respondents) were sent with a link to the online questionnaire. Those who could not be reached by email, paper-based questionnaires were distributed among them. The data collection period took place between March 2019 and October 2019.

The development of the questionnaire (Additional file 1) was adapted on the basis of detailed review of different literature and included four parts. The first part captured the demographic characteristics of the participants. The second part explored participants' attitudes toward factors pertinent for disclosure of IFs to research subjects using a 5-points Likert scale. The attitudes questions were adapted from Lohn et al. [14] and Berg et al. studies [14, 15]. Part three identified participants' perception of the duties to return IFs. The pertaining perception questions were developed based on the review of two internal genomic professionals and bioethicist of IFs management preference published in Ewuoso et al., (2016) [16]. The last part identified the barriers for disclosure of IFs which were adopted from Ramoni et al. [17]. For data analysis, participants' responses to strongly agree and agree were merged into one group (acceptance), and those who strongly disagree and disagree were combined into another group (non-acceptance). Prior to the main study work, questionnaire was validated by using face and content validity testing. The validity was conducted by 4 reviewers to assess questionnaire items for readability, clarity and comprehensiveness. The unclear items were amended, and the ineffective and nonfunctioning questions refined as advised of the reviewers. Cronbach's alpha for the attitudes scale was 0.805 0.769 for the perception scale, and 0.702 for the barriers scale indicating high internal consistency and reliability.

A descriptive analysis was used to describe the participants' characteristics and study outcomes as a percentage, frequencies, means and standard deviations. The study was approved by the institutional review board of King Fahad Medical City, and the completion of the questionnaire presumed participants' consent.

## Results

A total of 180 clinical genomic professionals in Saudi Arabia were invited to complete the questionnaire. Of whom, 113 respondents have completed the survey in its entirety for a 62% response rate. The mean age of respondents was 39.6 ± 8.00 years. The majority were male 69 (61.1%). The level of education and place of work varied, but the majority of respondents 66 (58.8%) were Ph.D. holders, and 78 (69%) indicated that they work in a health setting (Table 1).

**Table 1:** Participants' characteristics

Variables	n (%)
Age (mean ± SD)	39.6 ± 8.00
*Sex*	
Male	69 (61.1)
Female	44 (38.9)
*Level of education*	
Bachelor	13 (11.5)
Master	34 (30.1)
PhD	66 (58.4)
Years of experience (mean ± SD)	11.11 ± 10.00
*Affiliation*	
Health	78 (69)
Academic	35 (31)
*Country in which you received your most senior training*	
Saudi Arabia	41 (36.3)
USA	25 (22.1)
Canada	15 (13.3)
Europe	25 (22.1)
Others	7 (6.2)
*Guidelines on IFs available in your workplace*	
Yes	34 (30.1)
No	46 (40.7)
I do not know	33 (29.2)
*Did you encounter IFs in your research practice or by your colleague?*	
Yes	65 (57.5)
No	48 (42.5)
*Comfort in discussing IFs with research participants*	
Comfortable	82 (72.6)
Uncomfortable	31 (27.4)

When asked about the availability of guidelines governing reporting IFs, more than two-thirds of the respondents indicated they do not have or they do not know. However, 65 (57.5%) reported they encountered an IFs in their research practice or by their colleagues, and slightly more than a quarter felt uncomfortable in discussing the IFs with research participants.

Table 2 shows study participants' attitudes toward factors pertinent for disclosure of IFs to research subjects. About two-thirds of the study participants indicated research subject age and the anticipated psychosocial impact of IFs as factors may affect their decision of IFs disclosure to them. The majority of study participants 57 (75.9%) accepted that IFs should be returned to research subjects if they wanted them during informed consent.

**Table 2:** Attitudes toward factors associated with disclosure of incidental findings

	Strongly agree + agree	Neutral	Strongly disagree + disagree
Age of research participant	75 (66.4)	31 (27.4)	7 (6.2)
Psychosocial impact of the IFs	79 (69.9)	26 (23)	8 (8.1)
The test is analytically valid	84 (75.7)	21 (18.9)	6 (5.4)
The study participant wanted to receive the IFs during informed consent	57 (75.9)	12 (10.7)	13 (13.4)
*Severity of the condition*			
Serious and preventable/treatable^a^	97 (85.8)	9 (8)	7 (6.2)
Serious and not preventable/treatable^a^	65 (57.5)	30 (26.5)	18 (15.9)
Serious, late-onset and preventable/treatable^a^	92 (81.4)	14 (12.4)	7 (6.2)
Not-serious and preventable/treatable	92 (81.4)	13 (11.5)	8 (7.1)
Not-serious and not preventable/treatable	54 (48.2)	39 (34.8)	19 (17)
*Likelihood of disease threat*			
The chance < 1% (rare)	30 (27.3)	34 (30.9)	46 (41.8)
The chance 1–5% (few)	46 (41)	31 (27.7)	35 (31.2)
The chance 6–49% (some)	69 (62.7)	24 (21.8)	17 (15.5)
The chance ≥ 50% (most)	84 (80)	12 (11.4)	9 (8.6)
*Burden of intervention*			
Very low burden	40 (47.6)	34 (32.4)	21 (20)
Somewhat burdensome	59 (54.7)	37 (34.3)	12 (11.1)
Moderately burdensome	74 (69.2)	25 (23.4)	8 (7.4)
Highly burdensome	81 (75)	18 (16.7)	9 (8.3)

Not serious: not life threatening

^a^Serious: life threatening

Study participants were asked whether they accept to return the IFs based on the disease severity (serious vs. non-serious) and prevention/treatment, risk of disease threat occurrence, and burden of intervention for the disease. The majority of participants showed it was acceptable to return IFs in all categories of disease severity, even if the disease was preventable/treatable or not. As the likelihood of disease threat occurrence is increasing, more acceptance for disclosing IFs was reported. The majority 84 (80%) and 69 (62.7%) of the study participants indicated they would return the IFs if the risk of disease threat ≥ 50% and 6–49%, respectively. Moreover, three-quarters of the respondents accepted IFs if the burden of disease intervention was high.

Slightly more than two-thirds of the respondents accepted that IFs from genome studies should be made available to research participants, and about 81 (71.6%) reported that research subjects should have the choice of what IFs are disclosed to them. Moreover, 36 (31.9%) of the respondents perceived they could decide what IFs are disclosed to research participants. Remarkably, 36 (31.9%) indicated they could decide what IFs to be disclosed, and 36 (31.9%) reported they have no obligation to return IFs (Table 3).

**Table 3:** Study participants' perception of the duties to return IFs

	Strongly agree + agree	Neutral	Strongly disagree + disagree
IFs from genome studies should be made available to research participants?	77 (68.1)	23 (20.4)	13 (14.5)
Research participants should have a choice on what IFs are disclosed to them?	81 (71.6)	18 (15.9)	14 (12.4)
Research participants alone should make the decision on what IFs are disclosed to them?	61 (55)	27 (24.3)	23 (20.7)
I can decide what IFs are disclosed to research participants (e.g. only serious and treatable conditions)?	36 (31.9)	29 (25.7)	48 (42.4)
Research participants have the right to make decisions about receiving IFs if they have no prior knowledge or family history of the conditions listed?	61 (55)	27 (24.3)	23 (20.7)
I can override the research participant’s wishes if they consider it is not in their best interest to disclose a particular IF?	36 (31.9)	29 (25.6)	48 (42.5)
I can override the research participant’s wishes if they consider it is not in the best interest of their family members to disclose a particular IF?	35 (31)	34 (30.1)	44 (38.9)
I have no obligation to return Ifs	36 (31.9)	39 (34.5)	38 (33.6)

Moreover, our study participants reported a varied range of barriers to return IFs. Most viewed the uncertain clinical utility of genetic research results 93 (83%), need to use a clinically certified lab 75 (66.4%), the possibility that participants involved in genomic research will misunderstand the 73 (64.6%), the potential for emotional harm to participants (61%), the concern about adequacy of clinical follow-up 73 (64.6%), and the need to ensure access to trained clinician after disclosure of incidental findings (51%) as major barriers to the return of results (Table 4).

**Table 4:** Barriers to the return of IFs

Barriers	Major barrier	Minor barrier
Uncertain clinical utility of IFs	93 (83.0)	19 (17.0)
Possibility that participants will misunderstand IFs	73 (64.6)	40 (35.4)
Potential for causing emotional harm to the study participants	63 (55.8)	50 (44.2)
Need to ensure access to trained clinician after disclosure of IFs	70 (61.9)	43 (38.1)
Potential for loss of confidentiality	72 (63.7)	41 (36.3)
Possibility that association with IFs may not be valid	68 (60.7)	44 (39.3)
Need to use a clinically certified lab	75 (66.4)	38 (33.6)
Concern about adequacy of clinical follow-up	73 (64.6)	40 (35.4)
Potential to distort the line between research and clinical care	54 (47.8)	59 (52.2)
Possibility of social discrimination	65 (57.5)	48 (42.5)
Concern over liability for adverse outcomes of IFs disclosure	65 (57.5)	48 (42.5)
Time commitment required to return IFs	52 (46.0)	61 (54.0)
Possibility that genotyping may be inaccurate	75 (66.4)	38 (33.6)
Need to keep contact patients information update	56 (50.0)	56 (50.0)
Need to keep up to date with relevant associations of IFs with the disease	64 (56.6)	49 (43.4)
Cost of returning IFs to participants	64 (56.6)	49 (43.4)

## Discussion

There is an ongoing debate about the return of IFs in genomic research, and the recommendations for this keep evolving and challenging. In this study, we explored attitudes of clinical genomic professionals towards various factors surrounding the return of IFs from genomic research. Although more than half of the respondents reported experience with returning IFs, about one-quarter of respondents reported discomfort with the discussion of IFs with research participants. This observation indicates that clinical genomic professionals may be unprepared for the challenges posed by Next Generation Sequencing technology, which is identifying clinically relevant IFs more frequently [18, 19].

There was a high consensus that IFs should be reported irrespective of patient-specific and factors like patient’s age and psychosocial impact of the IFs. These attitudes align with the recommendations of the American College of Medical Genetics and Genomics signifying the importance of returning IFs without considering the patients' age, psychosocial status, and even their preferences [1]. Legitimate concerns about IFs pertaining to adult-onset disease provokes difficult issues and inflames debates especially in the lack of instructive data about the definite harms of knowing adult-onset diseases in children, or the real benefits to parents who might proactively take further actions to minimize or prevent the anticipated risk information generated from IFs.

In our study, there is tangible consensus in what clinical genomic professionals on the thresholds of returning IFs based on the severity and treatability of the diseases. The majority would consider the high relative risk of disease severity and clinical actionability. This indicates that the clinical genomic community holds a strong duty to offer IFs results to research participants and their family members, mainly if the findings are clinically preventable or treatable.

The majority of study respondents thought it was acceptable to disclose IFs information, even if the likelihood of the disease occurrence was low. As the likelihood increased, there was less reticence about this (i.e., the strongly disagree and disagree answers decreased, Table 2). This fits data reported by others where ethical reviews and empirical research that it is acceptable to return IFs regardless of the risk of disease occurrence [20–22].

Respecting research subject autonomy is one of the most fundamental ethical principles relating to research participation and returning incidental findings. Although the majority of the study respondents indicated that IFs from genome studies should be made available to research participants, about 36 (31.9%) reported they do not have the duty to return IFs. This conservative position is consistent with other scholars in the field who disagree that researchers do not have the obligation to return IFs [11, 12, 23]. Although those scholars’ views were contingent on the fulfillment of specific criteria, this could be seen as a paternalism approach to prevent the mythical Pandora felt after disclosing the harmful IFs [24].

The genomic information is not only affecting concerned individuals but may have a significant impact on the family, including offspring, and in some cases on their tribe. Besides, genomic information may have cultural significance for persons, entire family or tribe. Due to the complicated ethical and practical issues inherent in the disclosure of IFs, it is important to consider the nature of Saudi culture, religious beliefs, and societal structure when considering returning IFs. Saudi society composed of tribal and extended families and any disclosure of the confidential IFs may affect not just the patients, but would extend to the families and tribes. Such disclosure may create stigmatization and discrimination as dire social repercussions for the patient, entire family or tribe. In addition, the rate of consanguinity marriage in Saudi society is very high, and it is imperative to consider the consequences of not disclosing IFs especially in pediatric settings. The essence of the ethical dilemma surrounding IFs is maintaining the balance between the principles of patient autonomy in self-determination and nonmaleficence. Therefore, due consideration should be given to the sensitivity of genomic information and an appropriate system of disclosure of IFs should be established to provides an ethically elegant system to categorize all IFs disclosure acts that is consistent with the Islamic maxims and jurisprudence, Saudi culture, and align with international guidelines.

However, previous studies reported from Saudi Arabia revealed that Saudis’ see returning IFs as a moral duty and an ethical obligation [25, 26]. Hence, the criteria set forth by the by the ACMG recommendations of reporting only pathogenic or likely pathogenic variants of the listed 59 actionable genes need to be interpreted and implemented with cautions within the context of Saudi society [27].

The debate continues over how, when and who should return IFs to research subjects because of the complexity and scale of information in genomic research [28]. Thus, it becomes clear that an appropriate legislative measures and framework to give effect to the international law of human rights. Such framework should be in accordance with the countries’ social context and supported by education, research, training and public information. ACMG and NHLBI guidelines works as the international standardized bases for reporting of actionable information generated from clinical genomic sequencing. Both guidelines proposed the analytical/clinical validity, clinically actionable IFs, established and substantial risk of a serious health condition or reproductive importance, and patients desire to receive IFs as criteria for reporting IFs [13, 28]. In addition to these criteria, we uphold the inherent ethical and clinical principles outlining the ACMG and NHLBI criteria. Besides, we recommend synthesizing culturally sensitive pluses to assess the net effects and benefits of revealing the IFs to the participant, and every IF should be evaluated in a case by case conference [29].

Genomic IFs generated in clinical settings may have potential and direct clinical implications or utility for the patient and his/her family’s health, or useful for reproductive health or for future planning [30]. Therefore, the return IFs and access to treatment and follow up in clinical settings is an obligatory clinical duty. In a recent qualitative study from Saudi Arabia, participating researchers agreed on the importance of returning research results. However, some researchers indicated that returning research results is not the researcher’s duty or responsibility [26]. A significant proportion of the participants perceived that it is accepted to override the research participant’s wishes if they consider it in the best interest of their family members to disclose particular IFs. This breach of patients’ confidentiality and privacy creates legal issues in IFs management [8]. However, this perception is similar to William's (2012) study respondents (researchers and institutional review board (IRB) chairs) who indicated that family members should be informed if the disease is inheritable [31]. Moreover, a similar significant proportion of the participants perceived that it is accepted to override the research participant’s wishes if they consider it is not in their best interest to disclose particular IFs. Nevertheless, IRB chairs and members in studies conducted by Williams (2012), Simon (2011), and Dressler (2012) want researchers to predict IFs and explicitly state informed consent how they would manage IFs [31–33]. Remarkably, two studies showed that researchers, geneticists, and IRB chairs and members want serious and preventable IFs disclosed to the research participants irrespective of the subject's preference [30, 34].

An established clinical utility in certain circumstances constitutes a strong legitimate argument to prevail over the ethical imperative of non-disclosure. For instance, the clinical utility of IFs information like having a carrier status or a present risk or future disease risk for family members can override and disqualify the right of research subjects [22]. Major barriers including the uncertain clinical utility of IFs, IFs validity, and the possibility that participants might misunderstand disclosed IFs information are the most cited major barriers and are in line with previously reported findings [17, 20].

### Study limitations

The recruitment strategies, online and paper-based, were purposely intended to enable the gathering of a large national sample; nonetheless, the convenience sampling technique would never be considered representative of clinical genomic professionals in Saudi Arabia. Given that our study offers valuable evidence about the clinical genomic professionals’ attitudes and perception towards the deliberated disclosure of IFs, what clinical genomic professionals practice in the pragmatic situation might be not the same? Therefore, it is incredible to know how their practice is aligning with the reported findings until the experience if returning IFs is measured objectively. Although our results give the impression that clinical genomic professionals support the returning of IFs, this does not certainly mean that this is the norm to adopt within the Saudi context. But, other considerations or arguments that might impact on this matter should be collectively explored, for example, the appropriate use and limitations of limited health care or resources and difficulties in IFs interpretation, result in a different conclusion.

## Conclusions

In this study, there was a consensus to return clinically actionable IFs. Our study respondents support IFs disclosure, especially when the disease risk is increasing. Moreover, the matter of IFs is commonly encountered in the study respondents’ workplaces, and many places do not have yet guidelines governing IFs. Comprehensive guidelines must be established and customized culturally to determine how IFs should be managed in Saudi Arabia. Overall, it is ethically imperative to disclose IFs if the results are accurate, interpretable, and medically associated with the research participant’s health and wellbeing. Incidental findings can save the research participant’s life and change the standards of clinical care; thus, IFs should be managed with optimal ethical standards that maintain the balance between the research participants and their families' best of interest and the progress of the research enterprise. Implications of returning IFs, for genomic research participants and enterprise, need to be explored empirically within the Saudi context.

## Supplementary Information

**Additional file 1**. Study questionnaire.

## Data Availability

The datasets generated and/or analyzed during the current study are not publicly available due to our institutional policy but are available from the corresponding author on reasonable request.

## Abbreviations

IFs: Incidental findings
ACMG: American College of Medical Genetics and Genomics
NHLBI: National Heart, Lung, and Blood Institute
IRB: Institutional Review Board

## References

[1] Green RC, Berg JS, Grody WW, Kalia SS, Korf BR, Martin CL (2013). ACMG recommendations for reporting of incidental findings in clinical exome and genome sequencing. Genet Med.

[2] Wolf SM, Lawrenz FP, Nelson CA, Kahn JP, Cho MK, Clayton EW, Fletcher JG, Georgieff MK, Hammerschmidt D, Hudson K, Illes J (2008). Managing incidental findings in human subjects research: analysis and recommendations. J Law Med Ethics.

[3] Jackson L, Goldsmith L, O’connor A, Skirton H (2012). Incidental findings in genetic research and clinical diagnostic tests: a systematic review. Am J Med Genet Part A.

[4] Clarke AJ (2014). Managing the ethical challenges of next-generation sequencing in genomic medicine. Br Med Bull.

[5] Fernandez CV, Bouffet E, Malkin D, Jabado N, O’Connell C, Avard D, Knoppers BM, Ferguson M, Boycott KM, Sorensen PH, Orr AC (2014). Attitudes of parents toward the return of targeted and incidental genomic research findings in children. Genet Med.

[6] Bollinger JM, Green RC, Kaufman D (2013). Attitudes about regulation among direct-to-consumer genetic testing customers. Genet Test Mol Biomark.

[7] Harris A, Kelly SE, Wyatt S (2013). Counseling customers: emerging roles for genetic counselors in the direct-to-consumer genetic testing market. J Genet Counsel.

[8] Bledsoe MJ, Grizzle WE, Clark BJ, Zeps N (2012). Practical implementation issues and challenges for biobanks in the return of individual research results. Genet Med.

[9] Clayton EW, McGuire AL (2012). The legal risks of returning results of genomics research. Genet Med.

[10] Ossorio P (2012). Taking aims seriously: repository research and limits on the duty to return individual research findings. Genet Med.

[11] Lockhart NC, Yassin R, Weil CJ, Compton CC (2012). Intersection of biobanking and clinical care: should discrepant diagnoses and pathological findings be returned to research participants?. Genet Med.

[12] Ma’n HZ, Knoppers BM (2012). International normative perspectives on the return of individual research results and incidental findings in genomic biobanks. Genet Med.

[13] Bookman EB, Langehorne AA, Eckfeldt JH, Glass KC, Jarvik GP, Klag M, Koski G, Motulsky A, Wilfond B, Manolio TA, Fabsitz RR (2006). Reporting genetic results in research studies: summary and recommendations of an NHLBI working group. Am J Med Genet Part A.

[14] Lohn Z, Adam S, Birch P, Townsend A, Friedman J (2013). Genetics professionals' perspectives on reporting incidental findings from clinical genome-wide sequencing. Am J Med Genet Part A.

[15] Berg JS, Foreman AK, O’daniel JM, Booker JK, Boshe L, Carey T, Crooks KR, Jensen BC, Juengst ET, Lee K, Nelson DK (2016). A semiquantitative metric for evaluating clinical actionability of incidental or secondary findings from genome-scale sequencing. Genet Med.

[16] Ewuoso C (2016). A systematic review of the management of incidental findings in genomic research. BEOnline: J West Afr Bioethics Train Program..

[17] Ramoni RB, McGuire AL, Robinson JO, Morley DS, Plon SE, Joffe S (2013). Experiences and attitudes of genome investigators regarding return of individual genetic test results. Genet Med.

[18] Kohane IS, Hsing M, Kong SW (2012). Taxonomizing, sizing, and overcoming the incidentalome. Genet Med.

[19] Johnston JJ, Rubinstein WS, Facio FM (2012). Secondary variants in individuals undergoing exome sequencing: screening of 572 individuals identifies highpenetrance mutations in cancer-susceptibility genes. Am J Hum Genet.

[20] Middleton A, Morley KI, Bragin E, Firth HV, Hurles ME, Wright CF, Parker M (2016). Attitudes of nearly 7000 health professionals, genomic researchers and publics toward the return of incidental results from sequencing research. Eur J Hum Genet.

[21] Wright MF, Lewis KL, Fisher TC (2013). Preferences for results delivery from exome sequencing/genome sequencing. Genet Med.

[22] Christenhusz GM, Devriendt K, Dierickx K (2013). To tell or not to tell? A systematic review of ethical reflections on incidental findings arising in genetics contexts. Eur J Hum Genet.

[23] Wolf SM, Crock BN, Van Ness B, Lawrenz F, Kahn JP, Beskow LM, Cho MK (2012). Managing incidental findings and research results in genomic research involving biobanks and archived data sets. Genet Med.

[24] Townsend A, Adam S, Birch PH (2012). “I want to know what's in Pandora's box”: comparing stakeholder perspectives on incidental findings in clinical whole genomic sequencing. Am J Med Genet Part A.

[25] Aloraini T, Abdulrahim A, Karbani GA (2019). Attitudes of geneticists and patients toward incidental findings in Saudi Arabia. J Biochem Clin Genet.

[26] Alahmad G, Alzahrany H, Almutairi AF (2020). Returning results of stored biological samples and biobanks: perspectives of Saudi Arabian biomedical researchers. Biopreserv Biobank.

[27] Kalia SS, Adelman K, Bale SJ, Chung WK, Eng C, Evans JP, Herman GE, Hufnagel SB, Klein TE, Korf BR, McKelvey KD (2017). Recommendations for reporting of secondary findings in clinical exome and genome sequencing, 2016 update (ACMG SF v2. 0): a policy statement of the American College of Medical Genetics and Genomics. Genet Med.

[28] Fabsitz RR, McGuire A, Sharp RR, Puggal M, Beskow LM, Biesecker LG, Bookman E, Burke W, Burchard EG, Church G, Clayton EW (2010). Ethical and practical guidelines for reporting genetic research results to study participants: updated guidelines from a National Heart, Lung, and Blood Institute working group. Circ: Cardiovasc Genet.

[29] Al Shakaki A. Islamic bioethical discourse in incidental findings: research genetic context. Innov Glob Health Prof Educ. 2019:22–23. 10.20421/ighpe2019.01.07.

[30] Downing NR, Williams JK, Daack-Hirsch S, Driessnack M, Simon CM (2013). Genetics specialists' perspectives on disclosure of genomic incidental findings in the clinical setting. Patient Educ Couns.

[31] Williams JK, Daack-Hirsch S, Driessnack M, Downing N, Shinkunas L, Brandt D, Simon C (2012). Researcher and institutional review board chair perspectives on incidental findings in genomic research. Genet Test Mol Biomark.

[32] Simon CM, Williams JK, Shinkunas L, Brandt D, Daack-Hirsch S, Driessnack M (2011). Informed consent and genomic incidental findings: Irb chair perspectives. J Empir Res Hum Res Ethics.

[33] Dressler LG, Smolek S, Ponsaran R, Markey JM, Starks H, Gerson N, Lewis S (2012). Irb perspectives on the return of individual results from genomic research. Genet Med.

[34] Klitzman R, Appelbaum PS, Fyer A, Martinez J, Buquez B, Wynn J, Waldman CR, Phelan J, Parens E, Chung WK (2013). Researchers’ views on return of incidental genomic research results: qualitative and quantitative findings. Genet Med.

