# Supplementary material for: To disclose, or not to disclose? Perspectives of clinical genomics professionals toward returning incidental findings from genomic research
Source: BMC Med Ethics. 2021 Jul 27;22:101. doi: 10.1186/s12910-021-00670-y (PMC8314473; doi:10.1186/s12910-021-00670-y)
Supplement: Supplementary file 1 — Additional file 1. Study questionnaire. [file 12910_2021_670_MOESM1_ESM.docx]

**To disclose, or not to disclose? Perspectives of clinical genomics professionals toward incidental findings in context of Genetic Research**

On behalf of the study team, we are delighted to invite you to participate in a research study survey of Saudi genetic researchers about their attitudes and perception related to disclosure of incidental findings generated from genomic research.

Genetic testing has developed over recent years and a number of different tests are now available that are relatively quick and inexpensive to carry out. As testing becomes more detailed, the number of possible diagnoses identified will increase but also the possibility of finding other unrelated genetic information. These types of findings have been called Incidental Findings (IFs).

A number of recommendations about the return of research results have been published. This study will document and quantify how researchers are addressing these issues in the field, their attitudes and perception about outstanding challenges, and their preferences for additional guidance.

The Institutional Review Board at King Fahad Medical City has approved this research project. If you agree to take this survey, all of the information you provide will be kept in strictest confidentiality. No identity will be collected, therefore, we will not be able to identify individual responders or link individual identifying information to survey responses. Completion of this survey implies your consent to participate in this study. We anticipate that it will take about 20 minutes to complete the survey

Please feel free to contact the principal investigator for any clarification or

**Isamme AlFayyad, MA (BioEthics)**

Clinical Research Specialist

Research Center, KFMC

Tele: 011-288 9999 / 10848

**Background:** Clinical genomic professional and researchers are increasingly facing decisions about returning incidental findings (IFs) from genome research. Although previous studies have shown that research participants are interested in receiving IFs, Yet there has been argument about the extent of researcher obligation to return IFs. We aimed in this study to explore the attitudes of clinical genomics professionals toward disclosure of IFs, their perception of the duties to return IFs, and identifying the barriers for disclosure of IFs. **Methods:** a cross-sectional study was conducted between March 2019 and November 2019 using online line and paper-based self-administered questionnaire among clinical genomic professionals working in different academic and health care settings across Saudi Arabia. **Results:** Of 180 clinical genomic professionals, 113 (62%) responded. Sixty-five (57.5%) respondents had faced IFs in their practice and 31 (27.4%) were not comfortable in discussing IFs with their research subjects. Less than one-third of the respondents reported the availability of guidelines governing IFs. The majority of participants showed it was acceptable to return IFs in all categories of disease severity (range: 48.2%-85.8%). The majorities 84 (80%) and 69 (62.7%) of the study participants indicated they will return the IFs if the risk of disease threat ≥50% and 6-49%, respectively. More than two-thirds of the respondents accepted that IFs from genome studies should be made available to research participants (68.1%) and 36 (31.9%) reported they have no obligation to return IFs. Most viewed the uncertain clinical utility of genetic research results 93 (83%) as the utmost major barrier to the return IFs. **Conclusion:** clinical genomics professionals have positive attitudes and perception toward the returning IFs from genomic research, yet some revealed no duty to do so. Detailed guidelines must be established to provide insights into how genomics professionals should be handled IFs.

**Keywords:** Incidental findings; disclosure, genomic research, attitudes, Perception, Barriers, Saudi Arabia

| **Demographic Characteristics** |
| --- |
| - **Age: ………………… (years)** |
| - **Gender:**   Male  Female |
| - **Level of Education:**   Bachelor.  Master.  PhD  Post-doctoral fellowship |
| - **Affiliation:**   Health care setting.  Academic setting. |
| - **Guidelines on IFs available in your workplace:**   Yes  No  I do not know |
| - **Years in practice: …………………(years)** |
| - **Country in which you received your most senior training:**   Saudi Arabia.  USA  Canada  Europe  Others |
| - **Did you encounter an Incidental Findings in your research practice or by your colleague?**   Yes  No |
| - **Comfort in discussing genetic results with research participants:**   Very comfortable  Comfortable  Uncomfortable  Very uncomfortable |

- **Factors that should be used to determine whether incidental findings should be offered to research participants** *(please answer the below variable from strongly agree to strongly disagree)*
- *I think patients should be able to receive information about conditions that are*:

|  | **Strongly agree** | **Agree** | **Neutral** | **Neutral** | **Strongly disagree** |
| --- | --- | --- | --- | --- | --- |
| - **Age of research participant** |  |  |  |  |  |
| - **Psychosocial impact** |  |  |  |  |  |
| - **The test is analytically valid** |  |  |  |  |  |
| - **The study participant opted to receive the incidental findings during informed consent** |  |  |  |  |  |
| - **Seriousness of the condition:** |  |  |  |  |  |
| - *Life-threatening and preventable/treatable* |  |  |  |  |  |
| - *Life-threatening and* ***not*** *preventable/treatable* |  |  |  |  |  |
| - *Life threatening,* ***late-onset*** *and preventable/treatable* |  |  |  |  |  |
| - *Serious (****but not life threatening****) and preventable/treatable* |  |  |  |  |  |
| - *Serious (****but not life threatening****) and* ***not*** *preventable/treatable* |  |  |  |  |  |
| - **Likelihood of disease: What is the chance that a serious threat will occur** |  |  |  |  |  |
| - *The chance <1% (rare)* |  |  |  |  |  |
| - *The chance 1-5% (few)* |  |  |  |  |  |
| - *The chance 6-49% (some)* |  |  |  |  |  |
| - *The chance ≥50% (most)* |  |  |  |  |  |
| - **Burden of intervention** |  |  |  |  |  |
| - *Very low burden* |  |  |  |  |  |
| - *Somewhat burdensome* |  |  |  |  |  |
| - *Moderately burdensome* |  |  |  |  |  |
| - *Highly burdensome* |  |  |  |  |  |

- **Perception on incidental findings disclosure:**

| - **Incidental findings from genome studies should be made available to research participants?** | | | | |
| --- | --- | --- | --- | --- |
| Disagree | Agree | Neutral | Disagree | Strongly disagree |
| - **Research participants should have a choice on what incidental findings are disclosed to them?** | | | | |
| Disagree | Agree | Neutral | Disagree | Strongly disagree |
| - **Research participants alone should make the decision on what incidental findings are disclosed to them?** | | | | |
| Disagree | Agree | Neutral | Disagree | Strongly disagree |
| - **I can decide what incidental findings are disclosed to research participants (e.g. only serious and treatable conditions)?** | | | | |
| Disagree | Agree | Neutral | Disagree | Strongly disagree |
| - **Research participants have the right to make decisions about receiving incidental findings if they have no prior knowledge or family history of the conditions listed?** | | | | |
| Disagree | Agree | Neutral | Disagree | Strongly disagree |
| - **I can override the research participant’s wishes if they consider it is in their best interest to disclose a particular incidental finding?** | | | | |
| Disagree | Agree | Neutral | Disagree | Strongly disagree |
| - **I can override the research participant’s wishes if they consider it is in the best interest of their family members to disclose a particular incidental finding?** | | | | |
| Disagree | Agree | Neutral | Disagree | Strongly disagree |
| - **I have no obligation to return incidental findings** | | | | |
| Disagree | Agree | Neutral | Disagree | Strongly disagree |

- **Barriers to the return of individual genetic research results**
- *Please select which one of the below is considered as a major or minor barrier*

| **Barriers** | **Major barrier** | **Minor barrier** |
| --- | --- | --- |
| 1. Uncertain clinical utility of incidental findings |  |  |
| 1. Possibility that participants will misunderstand incidental findings |  |  |
| 1. Potential for causing emotional harm to the study participants |  |  |
| 1. Need to ensure access to trained clinician after disclosure of incidental findings |  |  |
| 1. Potential for loss of confidentiality |  |  |
| 1. Possibility that association with incidental findings may not be valid |  |  |
| 1. Need to use a clinically certified lab |  |  |
| 1. Concern about adequacy of clinical follow-up |  |  |
| 1. Potential to distort the line between research and clinical care |  |  |
| 1. Possibility of social discrimination |  |  |
| 1. Concern over liability for adverse outcomes of incidental findings disclosure |  |  |
| 1. Time commitment required to return incidental findings |  |  |
| 1. Possibility that genotyping may be inaccurate |  |  |
| 1. Need to keep contact patients information update |  |  |
| 1. Need to keep up to date with relevant associations of incidental findings with the disease |  |  |
| 1. Cost of returning incidental findings to participants |  |  |

**Discussion:**

There is an ongoing debate about the return of IFs in genomic research, and the recommendations for this keep evolving and challenging. In this study, we explored attitudes of clinical genomic professionals towards various factors surrounding the return of IFs from genomic research. Although more than half of the respondents reported experience with returning IFs, about one-quarter of respondents reported discomfort with discussion of IFs with research participants. This observation indicates that clinical genomic professionals may be unprepared for the challenges posed by Next Generation Sequencing technology, which is identifying clinically relevant IFs more frequently.

There was high consensus that IFs should be reported irrespective of patient-specific and factors like patient’s age and psychosocial impact of the IFs. These attitudes align with the recommendations of the American College of Medical Genetics and Genomics signifying the importance of returning IFs without considering the patients' age, psychosocial status, and even their preferences (Green et al., 2013). Legitimate concerns about IFs pertaining to adult-onset disease provokes difficult issues and inflames debates especially in the lack of instructive data about the definite harms of knowing adult-onset diseases in children, or the real benefits to parents who might proactively take further actions to minimize or prevent the anticipated risk information generated from IFs.

In our study, there is tangible consensus in what clinical genomic professionals on the thresholds of returning IFs based on the severity and treatability of the diseases. The majority would consider the high relative risk of disease severity and clinical actionability. This indicates that the clinical genomic community holds a strong duty to offer IFs results to research participants and their family members, particularly if the findings are clinically preventable or treatable.

The majority of study respondents thought it was acceptable to disclose IFs information, even if the likelihood of the disease occurrence was low. As the likelihood increased, there was less reticence about this (ie, the strongly disagree and disagree answers decreased, Table 2). This fits to data reported by others where ethical reviews and empirical research that it is acceptable to return IFs regardless of the risk of disease occurrence.

Respecting research subject autonomy is one of the most fundamental ethical principles relating to research participation and returning incidental findings. Although the majority of the study respondents indicated that IFs from genome studies should be made available to research participants, about 36 (31.9%) reported they do not have the duty to return IFs. This conservative position is consistent with other scholars in the filed who disagree that researchers do not have the obligation to return IFs. Although those scholars’ views were contingent to the fulfillment of certain criteria, this could be seen as a paternalism approach to prevent the mythical Pandora felt after disclosing the harmful IFs.

A significant proportion of the participants perceived that it is accepted to override the research participant’s wishes if they consider it in the best interest of their family members to disclose particular IFs. This breach of patients’ confidentiality and privacy create legal issues IFs management. However, this perception is similar to Williams (2012) study respondents (researchers and IRB chairs) who indicated that family members should be informed if disease is inheritable. Moreover, a similar significant proportion of the participants perceived that it is accepted to override the research participant’s wishes if they consider it is not in their best interest to disclose particular IFs. Nevertheless, IRB chairs and members in studies conducted by Simon (2011), Dressler (2012), and Williams (2012) want researchers to predict IFs and explicitly state informed consent how they would manage IFs. Remarkably, two studies showed that researchers, geneticists, IRB chairs and members want serious and preventable Ifs disclosed to the research participants irrespective of the subject's preference (Downing et al., 2013, Klitzman et al., 2013).

An established clinical utility in certain circumstances constitutes a strong legitimate argument to prevail over the ethical imperative of non-disclosure. For instance, the clinical utility of IFs information like having a carrier status or a present risk or future disease risk for family members can override and disqualify the right of research subjects. Major barriers including the uncertain clinical utility of IFs, IFs validity, and the possibility that participants might misunderstand disclosed IFs information are the most cited major barriers and are in line with previously reported findings.

*Study limitations*

The recruitment strategies, online and paper-based, were purposely intended to enable the gathering of a large national sample; nonetheless the convenience sampling technique would never be considered representative of clinical genomic professionals in Saudi Arabia. Given that our study offers valuable evidence about the clinical genomic professionals’ attitudes and perception towards the deliberated disclosure of IFs, what clinical genomic professionals practice in pragmatic situation might be not the same. Therefore, it is incredible to know how their practice is aligning with the reported findings, until the experience if returning IFs is measured objectively. Although our results give the impression that clinical genomic professionals support the returning of IFs, this does not mean necessarily that this is the norm to adopt within the Saudi context. But, other considerations or arguments that might impact on this matter should be collectively explored, for example, the appropriate use and limitations of limited health care or resources and difficulties in IFs interpretation, result in a different conclusion.

**Conclusions**

In this study, there was a general consensus to return clinically actionable IFs. Our study respondents support IFs disclosure, especially when the disease risk is increasing. Moreover, the matter of IFs is commonly encountered within the study respondents’ work places, and many places do not have yet guidelines governing IFs. Comprehensive guidelines must be established and customized to determine how IFs should be managed in Saudi Arabia. Overall, it is ethically imperative to disclose IFs if the results are accurate, interpretable, and medically associated to the research participant’s health and wellbeing. Incidental findings can possibly save the research participant’s life and change the standards of clinical care; thus, IFs should be managed with optimal ethical standards that maintain the balance between the research participant’s and their families best of interest and the progress of the research enterprise. Implications of returning IFs, for genomic research participants and enterprise, need to be explored empirically within Saudi context.

- Kohane IS, Hsing M, Kong SW. Taxonomizing, sizing, and overcoming the incidentalome. Genet Med 2012;14:399–404.
- Johnston JJ, Rubinstein WS, Facio FM, et al. Secondary variants in individuals undergoing exome sequencing: screening of 572 individuals identifies highpenetrance mutations in cancer-susceptibility genes. Am J Hum Genet 2012;91:97–108.
- Middleton A, Morley KI, Bragin E, Firth HV, Hurles ME, Wright CF, Parker M. Attitudes of nearly 7000 health professionals, genomic researchers and publics toward the return of incidental results from sequencing research. European Journal of Human Genetics. 2016 Jan;24(1):21-9.
- Wright MF, Lewis KL, Fisher TC et al: Preferences for results delivery from exome sequencing/genome sequencing. Genet Med 2013; 16: 442–447.
- Christenhusz GM, Devriendt K, Dierickx K: To tell or not to tell? A systematic review of ethical reflections on incidental findings arising in genetics contexts. Eur J Hum Genet 2013; 21: 248–255.
- Lockhart NC, Yassin R, Weil CJ, Compton CC. Intersection of Biobanking and Clinical Care: Should Discrepant Diagnoses and Pathological Findings Be Returned to Research Participants? Genet Med. Apr; 2012 14(4):417–23.
- Wolf, Susan M., Crock, Brittney N., Van Ness, Brian, Lawrenz, Frances, Kahn, Jeffrey P., Beskow, Laura M., Cho, Mildred K., et al. Managing Incidental Findings and Research Results in Genomic Research Involving Biobanks and Archived Data Sets. Genet Med. 2012; 14(4):23.
- Zawati MH, Knoppers BM. International Normative Perspectives on the Return of Individual Research Results and Incidental Findings in Genomic Biobanks. Genet Med. Apr; 2012 14(4): 484–9.
- Townsend A, Adam S, Birch PH et al: “I want to know what's in Pandora's box”: Comparing stakeholder perspectives on incidental findings in clinical whole genomic sequencing. Am J Med Genet Part A 2012; 158A: 2519–2525.
- Bledsoe MJ, Grizzle WE, Clark BJ, Zeps N. Practical Implementation Issues and Challenges for Biobanks in the Return of Individual Research Results. Genet Med. Apr; 2012 14(4):478–83.
- Williams JK, Daack-Hirsch S, Driessnack M, Downing N, Shinkunas L, Brandt D, Simon C. Researcher and Institutional Review Board Chair Perspectives on Incidental Findings in Genomic Research. Genet Test Mol Biomarkers. Jun; 2012 16(6):508–13.
- Simon CM, Williams JK, Shinkunas L, Brandt D, Daack-Hirsch S, Driessnack M. Informed Consent and Genomic Incidental Findings: Irb Chair Perspectives. J Empir Res Hum Res Ethics. Dec; 2011 6(4):53–67.
- Dressler, Lynn G., Smolek, Sondra, Ponsaran, Roselle, Markey, Janell M., Starks, Helene, Gerson, Nancy, Lewis, Susan, et al. Irb Perspectives on the Return of Individual Results from Genomic Research. Genet Med. 2012; 14(2):7.
- Downing NR, Williams JK, Daack-Hirsch S, Driessnack M, Simon CM. Genetics Specialists' Perspectives on Disclosure of Genomic Incidental Findings in the Clinical Setting. Patient Educ Couns. Jan; 2013 90(1):133–8.
- Klitzman R, Appelbaum PS, Fyer A, Martinez J, Buquez B, Wynn J, Waldman CR, et al. Researchers' Views on Return of Incidental Genomic Research Results: Qualitative and Quantitative Findings. Genet Med. Jun 27.2013.
- McGuire A, Caulfield T, Cho M: Research ethics and the challenge of whole-genome sequencing. Nat Rev Genet 2008; 9: 152–156.
- Christenhusz GM, Devriendt K, Dierickx K. To tell or not to tell? A systematic review of ethical reflections on incidental findings arising in genetics contexts. European Journal of Human Genetics. 2013 Mar;21(3):248-55.
- Ramoni RB, McGuire AL, Robinson JO, Morley DS, Plon SE, Joffe S. Experiences and attitudes of genome investigators regarding return of individual genetic test results. Genetics in Medicine. 2013 Nov;15(11):882-7.
- Middleton A, Morley KI, Bragin E, Firth HV, Hurles ME, Wright CF, Parker M. Attitudes of nearly 7000 health professionals, genomic researchers and publics toward the return of incidental results from sequencing research. European Journal of Human Genetics. 2016 Jan;24(1):21-9.
